# Mometasone furoate/formoterol combination therapy increases frequency of days/nights free of short-acting β_2_-agonist use

**DOI:** 10.1186/1710-1492-7-S2-A15

**Published:** 2011-11-14

**Authors:** AS Nayak, RA Nathan, EO Meltzer, SF Weinstein, H Nolte

**Affiliations:** 1 Sneeze, Wheeze & Itch Associates, Normal, IL, USA; 2 Asthma and Allergy Associates and Research Center, Colorado Springs, CO, USA; 3 Allergy and Asthma Medical Group and Research Center, San Diego, CA, USA; 4 Allergy & Asthma Specialists Medical Group, Huntington Beach, CA, USA; 5 Merck Research Laboratories, Kenilworth, NJ, USA

## Objective

An important asthma therapy goal is to limit short-acting β_2_-agonist (SABA) rescue medication use. We evaluated the effect of mometasone furoate/formoterol (MF/F) combination therapy on SABA use with data from 3 phase III studies.

## Methods

All studies enrolled subjects (≥12y) with persistent asthma not well controlled on inhaled corticosteroids (ICS): study 1 (n=746 subjects with moderate persistent asthma randomized to 26wk MF/F-100/10µg, MF-100µg, F-10µg, or placebo twice daily [BID]); study 2 (n=781 subjects with moderate persistent asthma randomized to 26wk MF/F-200/10µg, MF-200µg, F-10µg, or placebo BID); study 3 (n=728 subjects with severe persistent asthma randomized to 12wk MF/F-400/10µg, MF/F-200/10µg, or MF-400µg BID). All studies included a 2-3wk MF BID run-in: P04073 (100µg), P04334(200µg), P04431(400µg). Percentage of SABA-free days/nights was a predefined secondary endpoint in all studies.

## Results

In study 1, mean change in the percentage of SABA-free days/ nights from baseline period (day -7 to 1) to overall treatment period was significantly increased for MF/F-100/10µg (18%) vs F-10µg (10%) and placebo (0% *P*≤.003), and numerically increased for MF/F-100/10µg vs MF-100µg (14%). In study 2, change in SABA-free days/nights was significantly increased for MF/F-200/10µg (21%) vs MF-200µg (15%), F-10µg (11%), and placebo (5%; *P*≤.033). In study 3, change in SABA-free days/nights was significantly increased for MF/F-400/10µg and MF/F-200/10µg groups (25% increase for each) vs the MF-400µg group (13%; *P*<.001).

## Conclusion

MF/F treatment resulted in significant improvement in the frequency of SABA-free days/nights vs individual MF and/or F monotherapies in subjects with persistent asthma not well controlled on ICS therapy.

